# Consumer Perceptions of Brand Localness and Globalness in Emerging Markets: A Cross-Cultural Context

**DOI:** 10.3389/fpsyg.2022.919020

**Published:** 2022-07-11

**Authors:** Asif Ali Safeer, Yewang Zhou, Muhammad Abrar, Fang Luo

**Affiliations:** ^1^Business School, Huanggang Normal University, Huanggang, China; ^2^Lyallpur Business School, Government College University Faisalabad, Faisalabad, Pakistan

**Keywords:** brand globalness, brand attitude, brand localness, consumer behavioral intentions, consumer ethnocentrism

## Abstract

The globalization of markets and consumer behavior has changed dramatically in recent years. Similarly, global and local brands are facing many challenges in emerging markets. Thus, in this backdrop, this research is intended to examine the impact of consumer perceptions of brand localness and globalness on brand attitude in order to predict consumer behavioral intentions (purchase intention, price premium, and word of mouth) in cross-cultural emerging markets (China and Pakistan). Additionally, this research considered the moderating effects of consumer ethnocentrism and brand familiarity as a control variable. This study used an online survey to examine 1,562 responses from Chinese (*n* = 768) and Pakistani (*n* = 794) consumers regarding local and global brands. The proposed hypotheses were analyzed by using the partial least square-structural equation modeling method. The findings indicated that the consumer perceptions of brand localness and brand globalness had a substantial impact on brand attitude, which in turn favorably influenced consumer behavioral intentions in China and Pakistan. The brand attitude was a crucial mediator in both markets but was more critical in China than Pakistan. The interaction moderating effects of consumer ethnocentrism and consumer perceptions of brand localness positively influenced brand attitude in China, whereas consumer ethnocentrism and consumer perceptions of brand globalness negatively influenced brand attitude in Pakistan. Interestingly, brand familiarity was discovered a substantial control variable in both markets, except for purchase intention in Pakistan. This research contributed to Fishbein’s attitude theory and social identity theory. This research offers important recommendations to local and global marketers and brand managers in formulating and employing several positioning, market segmentation, and targeting strategies that may assist them in competing effectively in emerging markets.

## Introduction

The growing impact of globalization has had a profound effect on emerging markets. Similarly, the impact of globalization on numerous markets has gained many researchers’ attention to study the issues affecting consumers’ preferences, attitudes, and behaviors toward local and global brands (Diamantopoulos et al., 2019; Steenkamp, 2019). On the other side, many local and international companies are targeting consumers with the flood of various products and services in emerging markets. These products and services influenced consumers’ lifestyles and consumption ideologies (Liu et al., 2020). In these dynamic environments, consumers are increasingly adopting a critical attitude, as evidenced by several countries in South America, Asia, and the Middle East, which continue to enjoy significant growth with transformations and effectively fulfill consumer needs in their markets (Mandler et al., 2021). Similarly, emerging markets continue to expand more rapidly than developed markets. Emerging markets are defined as economies experiencing significant economic growth due to government policies aimed at economic liberalization (Paul, 2020). Similarly, emerging markets are increasingly acknowledged as a unique mix of business, economic, cultural, financial, legal, political, institutional, and social environments in which to evaluate, re-evaluate, and revive earned knowledge and wisdom about how the corporate world operates, prevalent theories and their supporting information, and new discoveries that will improve human welfare in all environments, including the developed, transition, developing, and poorest countries of the world (Kearney, 2012). Likewise, emerging markets, particularly China and Pakistan, are important, offer a wide range of opportunities, and have a great potential for future research (Mushafiq, 2021; Hertenstein and Alon, 2022).

According to Srivastava et al. (2020), with $30 trillion in annual expenditures, emerging markets are projected to account for 50 percent of the world’s population by 2025. These enticing prospects have led to the entry of numerous global brands into emerging markets (He and Wang, 2017). As a result of this transition, several multinational firms are investing extensively in emerging markets to strengthen their global brands and increase market dominance (Srivastava et al., 2021). Thus, emerging markets have tremendous potential for growth (Jiao et al., 2021; Xinglong and Antwi, 2022). Similarly, local brands are also active, posing stiff competition for global brands. Additionally, emerging markets are seeing a transformation in consumer behavior and consumption patterns, with domestic brands rapidly substituting international brands (Cambefort, 2020). Given this evolution, marketers need to understand how consumers perceive homegrown and international brands in emerging markets (Srivastava et al., 2020). On the other hand, the significance of consumer attitudes and behavior has increased in recent years. Thus, domestic and international market dynamics have been completely changed. Consequently, managers must develop effective branding strategies to understand consumers’ attitudes and behavioral intentions in emerging markets (Safeer et al., 2021a).

Recent research has revealed the importance of brand attitude (Ko et al., 2021) and argued that global brands have come under criticism for various reasons, including their brand attitude (Lopez-Lomelí et al., 2019b). Many scholars have also emphasized the importance of consumer perceptions of brands, urging additional research on consumer perceptions of brand localness (CPBL) and brand globalness (CPBG) and their impact on brand attitude (BAT) in emerging markets (Mandler, 2019; Srivastava et al., 2020). Similarly, consumer ethnocentrism (CET) has recently emerged as a highly emphasized concept, and several authors strongly advocated that it must be investigated further in the context of emerging markets (Halkias et al., 2016; Mandler, 2019; Srivastava et al., 2020). The worldwide consumer preference for brands has been linked to a construct, such as a consumer ethnocentrism. Ethnocentric consumers demonstrate national favoritism in their buying decisions because they believe imported products pose a substantial threat to the national economy’s employment and growth opportunities (Sharma, 2015). These ethical intentions support domestic products (Lee et al., 2017). Thus, CET has been postulated as a critical theoretical construct inextricably associated with the local and global brands that require immediate managerial attention when managing brands competing in various markets (Diamantopoulos et al., 2019).

The current body of knowledge is available on a limited scale in a single country and a cross-cultural context. For instance, CPBL and CPBG have been examined on retail patronage in China (Swoboda et al., 2012), behavioral intentions (single construct) in China (Xie et al., 2015), purchase intention in Austria (Halkias et al., 2016), and India (Srivastava et al., 2020) and brand loyalty in Trinidad and Tobago (Rambocas and Narsingh, 2022) by using various mediating concepts in a single country context. Similarly, prior research has examined the impact of CPBL and CPBG on purchase likelihood/intentions in the United States and South Korea (Steenkamp et al., 2003), Denmark, Turkey, and Singapore (Özsomer, 2012), Austria and Bulgaria (Sichtmann et al., 2019), Bosnia and Herzegovina and Slovenia (Kolbl et al., 2020), and price premium (PP) in South Korea and Germany (Mandler et al., 2021) through various mediating concepts in a cross-cultural context. Thus, the current literature highlights the gap by emphasizing consumer perceptions of brand localness and globalness in various environments, and researchers are urged to conduct additional studies on the topic (Liu et al., 2021), particularly using the brand attitude in emerging markets (Mandler, 2019; Srivastava et al., 2020). On the other side, previous research demonstrated that brand familiarity (BF) is an essential element that may affect brand attitude and consumer behavioral intentions (Halkias et al., 2016; Kolbl et al., 2020; Mandler et al., 2021). Thus, BF was considered an important control variable in this study. In this background, we propose new research in cross-cultural emerging markets (China and Pakistan) context to investigate the impact of CPBL and CPBG on predicting consumer behavioral intentions (PI, PP, WOM) via brand attitude while considering the moderating impact of CET and BF as a control variable. As a result, we address the following questions:

To what extent do consumers’ perceptions of brand localness and globalness influence brand attitude and consumer behavioral intentions (PI, PP, WOM) regarding local and global brands?

To what extent does CET act as a moderator between CPBL, CPBG, and brand attitude in emerging markets?

To what extent does brand attitude mediate the relationships between CPBL, CPBG, and consumer behavioral intentions in emerging markets?

Theoretically, this research is expected to contribute to Fishbein’s attitude theory for assessing consumer attitude and its subsequent impact on consumer behavior in emerging markets. Similarly, this study is expected to contribute to the social identity theory by examining the moderating effects of consumer ethnocentrism. Practically, this research is anticipated to reveal meaningful managerial implications by emphasizing the diverse mechanisms underlying how local and global brand preferences may increase CPBL and CPBG to improve brands’ evaluations in emerging markets. To organize this research, we first describe the significance of the topic, the research gap, and associated questions, then develop hypotheses supported by theory and literature. After that, we demonstrate the methodology, results, and discussions of the findings. Finally, we present the conclusion, implications, research limitations, and research agenda for the future.

## Theoretical Framework and Hypotheses Development

The proposed research model is supported by Fishbein’s attitude and social identity theories. Fishbein and Ajzen (1975) offered the most lucid explanation of the theoretical underpinning for attitudes. Attitude theory defines that the formation of an attitude is determined by the evaluation of an attribute, which is then amplified by the attribute’s strength of association with the object (Fishbein and Ajzen, 1975; Huber and Leone, 1979). Similarly, an individual’s attitudes are determined by his most prominent beliefs at a given time. Beliefs are the subjective associations between distinct notions. In a given context, salient beliefs are those triggered from memory and “considered” by the individual (Fishbein and Ajzen, 1975; Olson et al., 1979). The fundamental theoretical premise of Fishbein’s attitude theory is that beliefs create attitude. Since attitude is formed by a collection of salient beliefs, attitude modifications must be mediated by these beliefs. Therefore, in order to alter an individual’s attitude toward a concept, it is essential to change the individual’s core beliefs about that concept (Fishbein and Ajzen, 1975; Lutz, 1975). Thus, consumers’ perceptions about a brand’s attributes are of primary importance to marketing researchers, who have concentrated on the brand’s attitude to comprehend consumer behavior (Mitchell and Olson, 1981). As global and local brands are associated with several abstract concepts, such as brand attitude. A brand’s functional attributes serve consumer needs and positively influence consumer behavior by delivering cognitively oriented benefits (Punyatoya et al., 2014; Halkias et al., 2016). Thus, we predict that consumer perceptions of brand localness and globalness influence the local and global brands, which in turn influence consumer attitudes and behavioral intentions toward those brands. As a result, CPBL and CPBG are hypothesized to be linked with brand attitude, resulting in favorable consumer behavioral intentions (PI, PP, WOM).

**FIGURE 1:**
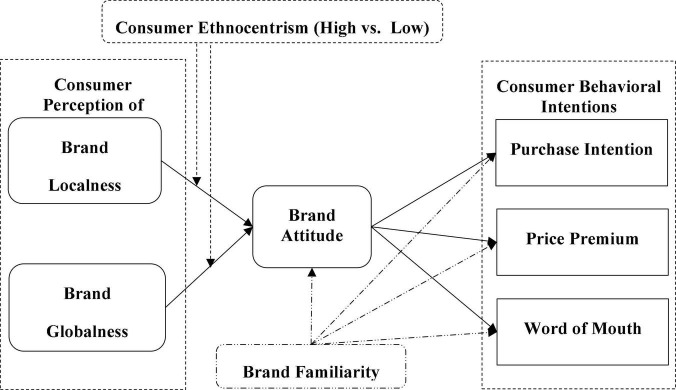
Proposed research model.

Social identity theory is described as “that part of an individual’s self-concept which derives from his knowledge of his membership in a social group (or groups) together with the value and emotional significance attached to that membership” (Tajfel, 1981, p. 255). Social identity theory is centered on prejudice, discrimination, and other situations that promote intergroup behavior (Hogg et al., 1995). Following in-group and out-group, individuals classify on a cognitive basis by identifying differences and similarities among groups with meaningful relationships, such as perceptions based on attitudes and behavior (Turner, 2010). From a global perspective, the home country is considered an in-group while the foreign country is an out-group (Zeugner-Roth et al., 2015). Social identity theory suggests that individuals strive to improve the status of their group. Ethnocentric consumers are the most likely to have a strong sense of “us” about home-grown versus international brands (Steenkamp et al., 2003). Consequently, ethnocentric consumers are more willing to embrace domestic market products’ advantages and minimize the advantages of foreign market products (Watson and Wright, 2000). Further, ethnocentric consumers tend to emphasize the Black and White distinction between in-group (domestic products) and out-group (foreign products) (Shankarmahesh, 2006). Thus, we predict that consumer ethnocentrism will have a positive effect on brand attitudes about local brands but a negative effect on attitudes toward global brands. Figure 1 illustrates the theoretical model and the subsequent hypotheses generated for China and Pakistan.

### Consumer Perceptions of Brand Localness, Brand Globalness, and Brand Attitude

The consequences of rapid globalization claimed that many multinational corporations launched aggressive marketing strategies for their international brands (Alden et al., 2006). However, some researchers suggested that international interdependence and interconnection do not refer to homogeneity and conformity (Ger, 1999; Holton, 2000); the local culture also influences consumer behavior (Samli, 1995). Building a link based on local culture could be one tactic to shape local brands’ perceptions (Ger, 1999). The local brand is described as an available brand within a particular geographic area (Dimofte et al., 2008). According to Eckhardt (2005), a brand is created for the home country consumers; or an iconic brand that is culturally connected with the domestic market. The CPBL refers to the consumer perceptions about the local brand. Thus, CPBL can be defined as the consumers’ perceptions as “a brand symbolizes the values, needs, and aspirations of the local country members” (Özsomer, 2012). Davvetas et al. (2016) defined that CPBL captures consumers’ perceptions, and based on those perceptions, a brand is manufactured using local resources. The perceptions of brands as locals also benefit from originality, authenticity, and adaptability according to local needs and expectations (Ger, 1999; Steenkamp and De Jong, 2010). Consumer perception of brand localness develops an association with native cultures that support building value for local brands (Özsomer, 2012). Such perceptions toward local brands can be more favorable in order to foster favorable consumer attitudes about them (Hoskins et al., 2021). Past research signifies the importance of brand attitude in different consumers’ settings and geographic environments. For example, Igelbrink (2020) revealed that CPBL favorably affected consumer brand attitude, indicating that local brands with local affinity were more positively perceived in developed markets. Local brands benefit from strong ties to local culture and communities, which helps them understand local consumer needs. These associations foster favorable sentiments regarding homegrown brands and result in beneficial consumer behavior (Halkias et al., 2016).

Steenkamp et al. (2003) defined CPBG as “consumers believe that a brand is marketed in multiple countries and recognized as global.” Several scholars defined the global brands based on consumer perceptions as per the following:

(1) Global product units with a central organizational structure through the use of the same brand name globally; applying the targeting and segmentation toward relatively similar interests and needs worldwide, and eventually a unique global identity (Douglas et al., 2001).

(2) Brands that provide global myth (signs of international ideals) with quality signals and promise the social responsibility (Holt et al., 2004).

(3) The wide availability of brands that enjoy global recognition across many markets (Dimofte et al., 2008). Thus, CPBG affects consumer brand attitudes, as numerous consumers favor global brands for their established worldwide image, quality, and prestige (Steenkamp et al., 2003). According to Davvetas et al. (2015), CPBG significantly impacted brand attitude in developed markets (Austria). Likewise, Halkias et al. (2016) demonstrated that strengthening consumer perceptions of brand globalness helps in developing positive brand attitudes in Austria. Punyatoya et al. (2014) discovered that global brands with extended names positively impacted consumer brand attitude from the Indian perspective. Thus, we can hypothesize:

**H1:** CPBL positively affects brand attitude in emerging markets.
**H2:** CPBG positively affects brand attitude in emerging markets.

### The Brand Attitude and Consumer Behavioral Intentions

The term “brand attitude” refers to consumers’ continual liking or disliking of a brand, as well as their overall evaluation of the brand (Wu Paul and Wang, 2011). Behavioral intentions are described as “the degree to which a person has formulated conscious plans to perform or not to perform some specified future behavior” (Westerbeek and Shilbury, 2003, p. 14). Behavioral intentions can be characterized as favorable or unfavorable toward products and services (Chen and Chen, 2010). Behavioral intentions could have consisted of purchase intentions (PI), positive word of mouth (WOM), and paying premium prices (PP; Zeithaml et al., 1996; Coudounaris and Sthapit, 2017). To know about consumer behavioral intentions (CBIs), business managers usually use purchase intention measures to introduce new products, demand forecasting, and make marketing mix decisions for offering in the relevant markets (Morwitz et al., 2007). While using CBIs measures, academic researchers and business managers can predict subsequent purchases of products and services. This concept is the foundation of numerous theoretical research models of consumer behavior (Naseem et al., 2015). For instance, Fishbein and Ajzen (1975) suggested that “If one wants to know whether or not an individual will perform a given behavior, the simplest and probably the most efficient thing one can do is to ask the individual whether he intends to perform that behavior.” Similarly, Bagozzi (1983) explained that “intentions constitute a willful state of choice where one makes a self-implicated statement as to a future course of action.” According to Warshaw (1980), consumer behavior research models usually indicate intentions as an intervening construct between choice and attitude, inferring that intentions overtake beliefs when behavior correlates. Marketing literature reported that behavioral intentions are generally associated with consumers or customers, also called consumer behavioral intentions (CBIs) and CBIs outcomes play an essential role in guiding firms to develop and implement various marketing strategies (Risitano et al., 2017). Behavioral intentions are commonly used in branding research. Many researchers use consumer behavioral intentions by measuring purchase intentions, positive WOM, and price premium (Risitano et al., 2017; Safeer et al., 2021b).

According to Steenkamp and De Jong (2010), consumers have varying attitudes toward domestic and international products across many categories. Özsomer and Altaras (2008) demonstrated that consumers’ favorable attitudes toward global brands might increase their willingness to purchase. Ger (1999) asserted that domestic products could foster positive brand attitudes and impact consumers’ buying intentions by fulfilling local requirements. According to Dimofte et al. (2008), the positive consumer tendency toward global brands (by explicating its affective components) generates positive consumer feelings toward the preferences for global brands. Thus, global brands appreciate consumers’ positive word of mouth, ranging from favorable brand attitude to increasing their willingness to pay premium prices for the global brands (Davvetas et al., 2015). Additionally, Chaudhuri and Holbrook (2001) argued that positive brand evaluations should translate into a brand’s favorable consumer behavioral intentions. As a result, it is predicted that a favorable brand attitude will subsequently translate into favorable consumer behavioral intentions by stimulating their purchase intentions (Halkias et al., 2016). Likewise, local brands can also increase purchase intentions in developed and emerging markets by stimulating consumer attitudes (Özsomer, 2012; Swoboda et al., 2012). Fishbein and Ajzen (1975) demonstrated that attitude theory influences the attitudes of individuals, which in turn shapes their intentions and behavior. As a result, we can hypothesize:

**H3:** Brand attitude positively affects consumer behavioral intentions such as PI **(H3a)**, PP **(H3b)**, and WOM **(H3c)** in emerging markets.

### The Moderating Impact of Consumer Ethnocentrism

Consumer ethnocentrism is “the beliefs consumers hold about the appropriateness, indeed morality, of purchasing foreign-made products” (Shimp and Sharma, 1987). Similarly, ethnocentric customers prefer homegrown products over imported ones because they believe that purchasing native products strengthens their economy, which is beneficial to them (Sharma, 2015). Additionally, ethnocentric consumers believe that adopting imported products is an unpatriotic act and poses a considerable threat to the domestic economy, resulting in job losses for many people. As a result, individuals develop unfavorable attitudes toward imported goods (Kaynak and Kara, 2002). Thus, the effects of the CPBL and CPBG on brand attitude are contingent upon consumers’ ethnocentric level. For example, consumers with a high level of ethnocentricity believe that their domestic products are authentic, have cultural influence, and have strong associations with the products. They develop favorable attitudes toward domestic brands while developing negative attitudes and adopting a rigid purchasing behavior toward global brands (Steenkamp et al., 2003). On the other side, consumers with a low level of ethnocentricity place a high value on the status, prestige, and high quality of global brands (Vuong and Khanh Giao, 2020). Social identity theory posits that individuals aspire to enhance their group’s standing. Thus, ethnocentric consumers have a more favorable perception of domestic products (in-group) and enjoy the advantages of domestic products while developing prejudiced attitudes toward imported products (out-group) in order to reduce the advantages of foreign market products (Watson and Wright, 2000; Shankarmahesh, 2006). In light of this background, we hypothesize:

**H4:** CET exerts a positive moderating effect on the link between CPBL and brand attitude. This favorable association is stronger for high ethnocentric customers but weaker for low ethnocentric consumers in emerging markets.
**H5:** CET exerts a negative moderating effect on the link between CPBG and brand attitude. This negative association is stronger for high ethnocentric customers but weaker for low ethnocentric consumers in emerging markets.

### The Mediating Role of Brand Attitude

Prior research highlighted the importance of brand attitude in the local and global branding perspectives. According to Batra et al. (2000), perceived non-localness origin brands positively influence consumer attitudes toward global brands in emerging markets (India) and wish to see these brands as local. Thus, CPBG increases consumer attitudes toward global brands. Similarly, Winit et al. (2014) found that brand globalness positively impacted brand attitudes that directly influenced positive purchase intentions. Therefore, irrespective of a local or global brand, having a global image may favorably influence consumer attitudes and behavioral intentions. Consumers consider global brands more positively due to their high quality and global reach around the world, their globalness positively affect brand attitudes which influence them to pay higher prices for the global brands in Austria (Davvetas et al., 2015). According to Naseem et al. (2015), favorable global brand attitude may favorably impact consumer behavioral intentions toward local and global brands.

Consumers are always associated with global brands because of their best quality, higher prestige, and symbolic and functional benefits (Swoboda et al., 2012). These consumers’ associations with global brands considered global brands can fulfill consumer demands at local and global levels (Holt et al., 2004). These consumer perceptions assist global brands, which gain consumer confidence through a positive attitude that results in favorable behavioral intentions toward global brands (Halkias et al., 2016). In the same way, local brands are also considered an icon of local culture and provide benefits of quality and prestige (Steenkamp et al., 2003; Özsomer, 2012), which help consumers in developing a positive attitude and consumer behavioral intentions toward that brand.

Halkias et al. (2016) found that perceived brand localness and perceived brand globalness positively impacted brand attitude that significantly led to consumer behavioral intentions (purchase intentions) in Austrian (European) perspectives. Recent research also revealed that local and global brands favorably impacted consumer attitudes due to their excellent quality, positive image, and familiarity and significantly influenced consumers’ purchase likelihood (Lopez-Lomelí et al., 2019b). Similarly, local and global brand evaluation based on attitudes and purchase intentions helps companies segment their markets and consumers effectively (López-Lomelí et al., 2019a). In this background, we predict that CPBL and CPBG may positively impact brand attitudes that may influence consumer behavioral intentions (PI, PP, WOM) toward the preference of local and global brands in emerging markets. In this situation, brand attitude may perform an influential role as a mediator in emerging markets, particularly in China and Pakistan. Therefore, this study develops the following hypotheses:

**H6:** Brand attitude positively mediates the relationships between CPBL and consumer behavioral intentions, including PI (6a), PP (6b), and WOM (6c) in emerging markets.
**H7:** Brand attitude positively mediates the relationships between CPBG and consumer behavioral intentions, including PI (7a), PP (7b), and WOM (7c) in emerging markets.

### Brand Familiarity as a Control Variable

According to Kent and Allen (1994), “Brand familiarity reflects the degree of direct and indirect experiences of a consumer with a brand.” Campbell and Keller (2003) defined brand familiarity (BF) as the knowledge that consumers acquired from brands, and through brand association, these consumers save the brand’s knowledge in their memory. The brand’s knowledge can be acquired through advertising exposure, WOM communication, product trials, consumption, and interaction with salespeople (Tam Jackie, 2008). Many researchers have agreed that familiarity affects consumer behavior and decision-making (Tam Jackie, 2008). Thus, brand familiarity (BF) is a vital control variable when assessing the consumers’ perceptions toward perceived local and global brands (Steenkamp et al., 2003). Prior research also acknowledged that brand familiarity might influence brand attitude and consumer behavioral intentions (PI, PP, WOM) (Xie et al., 2015; Halkias et al., 2016). As a result, BF was included as a control variable in this study to examine the relationships between CPBL, CPBG, brand attitude, and consumer behavior intentions (PI, PP, WOM) in emerging markets.

## Materials and Methods

Despite the fact that the two countries (China and Pakistan) have significant religious, ethnic, cultural, and economic disparities (Waheed and Zhang, 2020), this study was undertaken in two emerging markets. According to World Meter (2021), China is the world’s most populous country, while Pakistan is the fifth most populous country. Moreover, this research lacks in these emerging markets, and several authors suggested examining the impacts of CPBL and CPBG on brand attitude and consumer behavior in emerging markets, such as China and Pakistan (Mandler, 2019; Srivastava et al., 2020). Due to cultural and ethnic diversity, consumers have a wide range of preferences for local and global brands in these countries (Akram et al., 2011; Xie et al., 2015).

The product categories and brands were chosen in both countries through focus group discussions. In accordance with the research objectives, we selected paired local and global brands across three product categories, namely fast food, shoes, and apparel, depending on consumers’ brand familiarity with the brands (Özsomer, 2012; Mandler et al., 2021). In China, the paired (local/global) brands were fast food: Real Kungfu/McDonald, shoes: Li-Ning/Nike, and apparel: Septwolves/Levis. Similarly, in Pakistan, the paired (local/global) brands were fast food: Fri-Chicks/McDonald, shoes: Servis/Nike, and apparel: ChenOne/Levis. For generalizing the findings, this study followed durable and non-durable as well as similar global brands to minimize the specific brand effects in both emerging markets. Each participant was exposed to three product categories and rated a pair (local/global) of brands in that category (Steenkamp et al., 2003; Mandler et al., 2021).

### Data Collection Method

This research applied the survey technique for data collection from the intended consumers of China and Pakistan. Sekaran and Bougie (2016) described a survey as “a system for collecting information from or about people to describe, compare, or explain their knowledge, attitudes, and behavior.” The survey method allows the researchers to test the theoretical models and hypotheses drawn from the literature (Bhattacherjee, 2012). The email survey has been widely employed by various researchers in developed, emerging, and cross-cultural contexts to determine consumers’ perceptions of brand localness and globalness (Steenkamp et al., 2003; Özsomer, 2012; Kolbl et al., 2019) because email surveys facilitate the researchers (for timely data collection) and respondents (to provide information on the proposed questionnaire) in an electronic environment (Mandler et al., 2021). In this background, the email survey method was the best fit and appropriate for this research.

The structured questionnaire was designed both in English and Chinese language. The English version of the questionnaire was circulated in Pakistan. While the same questionnaire was translated into the Chinese language and distributed in China. The questionnaire included only brand names and did not include the brand logos. The questionnaire was posted online and organized using a leading Chinese survey website, https://www.wenjuan.com. This study utilized well-known (seven-point) scales. For example, the construct of CPBL was modified from Swoboda et al. (2012), CPBG and CET were modified from Steenkamp et al. (2003). The brand attitude was taken from Halkias et al. (2016). Consumer behavioral intentions (PI, PP, WOM) were derived from previous research (Zeithaml et al., 1996; Price and Arnould, 1999), and BF was used from Xie et al. (2015).

For the initial pilot study, a total of 150 responses were received from Chinese and 130 responses from Pakistani consumers. After data reviewing and screening, 110 replies from Chinese and 96 responses from Pakistani consumers were analyzed. Using SmartPLS software, the results revealed that composite reliability (CR) values in China ranged from 0.81 to 0.96, and the CR values in Pakistan were between 0.78 and 0.94. As a result, it demonstrated that both cultures validated the constructs and had a firm grasp on the questions. After assessing pretesting results, we employed the non-probability (convenience) sampling approach to collect data from the intended large consumer population. Prior research related to consumers’ perceptions of brand localness and globalness has commonly used the non-probability sampling method, and it was the best fit technique to fulfill the research objectives (Davvetas et al., 2015; Davvetas and Halkias, 2019; Kolbl et al., 2019; Sichtmann et al., 2019). Similarly, the convenience sampling method is frequently applied in consumer behavior and marketing research (Safeer et al., 2021a), and it is the most effective way to collect information quickly and timely manner (Sekaran and Bougie, 2016).

It is essential to decide the sample size before data collection. However, this is very complex due to different considerations (Bryman, 2016). These considerations could be research design such as qualitative or quantitative, number of constructs used in research, target population, time constraints, budget limitations, statistical analysis, confidence interval, and generalization of results (Hair et al., 2019). Therefore, a larger sample is generally suitable for quantitative research (Bryman, 2016). The sample size may be calculated on the researcher’s assessment, or a mathematical formula can be used by following a table (Cohen et al., 2017). Krejcie and Morgan (1970) introduced a table widely used by researchers to calculate a given population’s sample size. Accordingly, the sample size must be 384 with a 95% confidence level and at 5% confidence interval by following that table. Hair et al. (2011) recommended that it is vital to consider data characteristics and model complexity while computing the PLS-SEM sample size. Further, Hair et al. (1998) suggested that a sample size between 300 and 500 is considered the best fit for variance-based structural equation modeling analysis. Considering all statistical methods and after pretesting, this research collected data from 480 consumers (*n* = 960 responses from each country and a total of *n* = 1,920 responses) from China and Pakistan. The methodology suggested collecting sample data in pairs (global-local brand) to evaluate consumer perceptions in emerging markets (Steenkamp et al., 2003; Özsomer, 2012; Mandler et al., 2021). It indicates that the sample size qualified for all the statistical and prior research requirements. After a rigorous assessment, filtering, and removing biased responses, 1,562 responses, including 768 responses from 384 Chinese consumers and 794 responses from 397 Pakistani consumers, were accepted for data analysis.

This study’s sample composition (male vs. female) was almost equal in China’s context. However, this composition revealed that male participation was higher than female in Pakistan because of a Muslim country. Further, mainly students (who are young and well-educated) participated in this study because students are brand savvy, fashion-conscious and at the forefront of globalization, making them a highly attractive segment for many companies (Mandler et al., 2021). The demographics of the consumers are summarized in Table 1.

**TABLE 1:** Participants’ profile.

Type	China	Pakistan
**Sample size (final responses)**	768	794
**Gender**	%	%
Male	51.3	76.7
Female	48.7	23.3
**Age**		
20–27	84.9	81.49
28–35	11.07	9.82
36–43	4.04	8.69
**Education**		
High school	1.56	2.9
Bachelor	39.71	36.65
Master	47.53	45.72
Doctoral	10.42	11.34
Other professional degree	0.78	3.4
**Profession**		
Students	86.33	76.45
Government/Public organization sector officials	5.86	11.34
Private business sector officials	7.29	6.93
Self-employed/Unemployed	0.52	5.29
**Monthly family income (USD)**		
Up to $1,000	30.73	52.77
1,001 to $1,500	25.78	20.65
1,501 to $2,000	14.71	11.34
2,001 to $2,500	12.5	4.41
2,501 to $3,000	3.65	2.39
More than $3,000	12.63	8.44

## Results

Partial least square (PLS) structural equation modeling (SEM) is extensively used in many social sciences disciplines, including marketing and consumer behavior (Hair Joseph et al., 2019). Likewise, PLS is a causative prediction SEM technique that forecasts statistical models by associating their structure with a causal explanation (Sarstedt et al., 2017). PLS is also a robust multivariate method for examining complex research issues incorporating unobserved variables and complex multi-faceted interactions between distinct constructs. According to Hair Joseph et al. (2019), PLS-SEM performs very well with a larger sample size and is better for regression analysis to determine mediation effects.

### Measurement Model Assessment

According to Hair et al. (2018), it is critical to analyze CR to examine internal consistency, indicators reliability, and evaluation of average variance extracted (AVE) to determine the reflective model’s convergent validity. Further, Hair et al. (2018) added that indicators with values between 0.70 and 0.90 should be deemed more suitable. Using the PLSc technique, we discovered that all outer loadings were within an acceptable range according to cutoff criteria (see Table 2). Similarly, CR, AVE values, and factor loadings were all within an acceptable range (Hair et al., 2011, Hair et al., 2018) in China and Pakistan.

**TABLE 2:** Construct reliability and validity values.

Variable	Items	China data (*n* = 768)			Pakistan data (*n* = 794)		
		Factor loadings	CR	AVE	Factor loadings	CR	AVE
BF	BF1	0.82	0.82	0.7	0.88	0.85	0.74
	BF2	0.85			0.84		
CPBG	CPBG1	0.86	0.86	0.68	0.84	0.87	0.69
	CPBG2	0.76			0.85		
	CPBG3	0.86			0.81		
CPBL	CPBL1	0.75	0.79	0.57	0.88	0.88	0.71
	CPBL2	0.81			0.84		
	CPBL3	0.7			0.81		
BAT	BAT1	0.79	0.88	0.71	0.8	0.87	0.7
	BAT2	0.9			0.84		
	BAT3	0.85			0.86		
PI	PI1	0.8	0.88	0.7	0.85	0.89	0.74
	PI2	0.87			0.86		
	PI3	0.85			0.86		
PP	PP1	0.91	0.89	0.74	0.89	0.89	0.74
	PP2	0.86			0.85		
	PP3	0.82			0.84		
WOM	WOM1	0.81	0.87	0.7	0.84	0.88	0.71
	WOM2	0.88			0.84		
	WOM3	0.82			0.86		
CET	CET1	0.64	0.85	0.66	0.88	0.82	0.61
	CET2	0.71			0.79		
	CET3	0.9			0.85		
	CET4	0.81			0.69		

To determine the discriminant validity, our results satisfied the Fornell and Larcker (1981) measure which evaluates that the construct’s square root of the AVE values should be greater than the correlations with other constructs (Hair et al., 2018). Henseler et al. (2015) later proposed the HTMT ratio as a criterion. Our results also met the criterion (see Table 3), indicating that the correlations between constructs should be less than 0.90. As a result, the discriminant validity of the HTMT ratio method has also been established.

**TABLE 3:** Heterotrait-monotrait ratio (China/Pakistan).

	1	2	3	4	5	6	7	8
1. BAT								
2. BF	0.76/0.71							
3. CET	0.13/0.54	0.24/0.51						
4. CPBG	0.62/0.72	0.73/0.74	0.45/0.41					
5. CPBL	0.39/0.71	0.23/0.62	0.53/0.65	0.11/0.57				
6. PI	0.78/0.83	0.74/0.69	0.17/0.55	0.61/0.70	0.24/0.68			
7. PP	0.63/0.76	0.62/0.72	0.10/0.52	0.54/0.72	0.27/0.66	0.72/0.80		
8. WOM	0.71/0.78	0.68/0.71	0.09/0.53	0.60/0.71	0.29/0.72	0.77/0.80	0.81/0.82	

### Cross-National Invariance Measurement

Before comparing results between China and Pakistan, it is critical to consider cross-national measurement invariance. Steenkamp et al. (2003) proposed that all brand attribute measurements should be evaluated across groups before performing SEM multi-group analysis. Hult et al. (2008) demonstrated that when estimating constructs, a lack of measurement equivalence between groups might result in measurement errors, which can lead to misleading conclusions. Following PLS-SEM, Henseler et al. (2016) presented a three-step measurement invariance of composite models (MICOM) approach for determining invariance. According to Henseler et al. (2016), the MICOM method can be applied to examine reflective and formative models using PLS-SEM. Thus, previous research widely employed the MICOM method on reflective models in order to measure invariance between groups (Rasoolimanesh et al., 2017; Cheah et al., 2019; Zhang et al., 2019; Al-Swidi et al., 2021; Rastegar et al., 2021).

Step 1: Configural Invariance is an essential requirement for all group analyses. It implies that the indicators, data processing, algorithm settings, and applications should be similar across groups. This study met the first step by assuring that indicators, data processing, algorithms settings, and applications were the same across groups.

Step 2: Compositional Invariance is established when the original correlation value is higher than 5% (Henseler et al., 2016). We performed permutation analysis at a two-tailed 0.05 significance level using 5000 bootstrapped subsamples. The results exhibited a significant difference between the two groups in terms of CPBL3 outer loadings. We removed the indicator CPBL3 and discovered that all results met the recommended criteria with a *p*-value greater than (>) 0.05 (see Table 4). The absence of the indicator had no effect on the results or interpretations of reflective models (Henseler et al., 2016). The establishment of step 1 and step 2 indicates that partial invariance has been established. Thus, structural relationships between groups can be analyzed.

**TABLE 4:** Compositional invariance results (China and Pakistan).

Construct	Original correlation	Correlation permutation mean	5.0%	Permutation *P*-values
BAT	1.000	1.000	1.000	0.309
BF	1.000	1.000	1.000	0.186
CET	0.999	0.999	0.998	0.248
CPBG	1.000	1.000	1.000	0.260
CPBL	1.000	1.000	1.000	0.251
PI	1.000	1.000	1.000	0.072
PP	1.000	1.000	1.000	0.296
WOM	1.000	1.000	1.000	0.192

Step 3: If the mean values and variances are within the range of 2.5–97.5%, with a *p*-value greater than 0.05. It denotes that complete invariance has been established. This research revealed differences in the third stage and determined that mean values have not achieved the threshold; while only one value of variances achieved the threshold, others surpassed it (see Table 5). As a result, this study did not achieve full invariance. However, partial invariance allows assessing construct relationships across groups (Henseler et al., 2016).

**TABLE 5:** Equivalence of mean value and variances results (China and Pakistan).

Construct	Mean – original difference China – Pakistan	Mean – permutation mean difference China – Pakistan	2.5%	97.5%	Permutation *P*-values	Equivalence of mean value
BAT	−0.564	−0.003	−0.104	0.099	0.028	No
BF	−0.291	−0.003	−0.103	0.088	0.015	No
CET	−1.132	−0.002	−0.101	0.104	0.039	No
CPBG	−0.369	−0.002	−0.102	0.093	0.026	No
CPBL	−0.642	−0.003	−0.103	0.096	0.031	No
PI	−0.492	−0.001	−0.099	0.091	0.027	No
PP	−0.966	−0.002	−0.096	0.096	0.037	No
WOM	−0.716	−0.003	−0.109	0.091	0.034	No

**Construct**	**Variance – original difference China – Pakistan**	**Variance – permutation mean difference China – Pakistan**	**2.5%**	**97.5%**	**Permutation *P*-values**	**Equivalence of variance value**

BAT	−0.335	−0.002	−0.155	0.142	0.018	No
BF	−0.312	−0.003	−0.158	0.156	0.015	No
CET	0.321	0.001	−0.090	0.093	0.015	No
CPBG	−0.411	−0.005	−0.144	0.134	0.019	No
CPBL	0.162	0.000	−0.102	0.102	0.002	No
PI	−0.043	−0.004	−0.143	0.142	0.586	Yes
PP	−0.244	−0.001	−0.127	0.134	0.003	No
WOM	−0.228	−0.001	−0.148	0.142	0.005	No

### Structural Model Assessment

The structural model evaluation process comprises determining the model’s collinearity, the relevance and significance of relationships, the coefficient of determination (R^2^), model fit, and the predictive relevance of the model (Hair et al., 2018). Collinearity must be evaluated before assessing structural relationships to ensure that it does not bias the regression outcomes. This study discovered that all VIF values were less than 3 (Hair Joseph et al., 2019), which assured no biases and multicollinearity in the data. Thus, structural relationships can be evaluated. We applied multi-group analysis with bootstrapping 5000 subsamples two-tailed at the 0.05 significance level to test the hypothesized relationships (Hair et al., 2012). After adjusting for BF effects, we observed that CPBL had a substantial effect on brand attitude in China. However, this effect was positive and slightly lower in Pakistan. Thus, hypothesis H1 was supported. On the other side, CPBG was strongly positive on brand attitude in Pakistan. However, this effect was positively significant but slightly lower in China than in Pakistan. Thus, hypothesis H2 was also supported. H3a–H3c found that brand attitude positively influenced consumer behavioral intentions, including PI, PP, and WOM, in both emerging markets. Therefore, H3a–H3c were supported. The controlling effects of BF positively impacted brand attitude and PI, PP, and WOM in China. Similar effects were reported in Pakistan except for PI. Thus, it demonstrates that BF did not influence the PI of Pakistani consumers.

According to H4, the interaction moderating effects of CET and CPBL were significantly positive on brand attitude in the Chinese context but had no effects in Pakistan. Thus, H4 was partially supported. H5 discovered that CET and CPBG had negatively significant on brand attitude in Pakistan but had no effects in China. As a result, H5 was also partially supported. To examine the mediation effects, this study found that the direct effects of CPBL were non-significant on Chinese consumers’ behavioral intentions (PI, PP, WOM). Thus, full mediation was reported in China. However, CPBL directly affected Pakistani consumers’ behavioral intentions (PI, PP, WOM), and partial mediation was found in Pakistan. Accordingly, H6a–H6c were supported. Similarly, the direct effects of CPBG were found positively significant on consumer behavioral intentions (PI, PP, WOM) in Pakistan, indicating partial mediation. In contrast, CPBG was observed non-significant direct effect on PI in China, indicating complete mediation. While CPBG discovered significant direct effects on PP and WOM in China, indicating partial mediation. Therefore, the positive mediation influence of brand attitude demonstrates that the hypotheses H7a–H7c were supported (see Table 6).

**TABLE 6:** Hypotheses results.

		China		Pakistan			
Hypotheses		β	*t*-value	β	*t*-value	Results support	
**Direct relationships**							
H1	CPBL - > BAT	0.36***	6.10	0.34***	5.70	Yes	
H2	CPBG - > BAT	0.26***	3.84	0.32***	4.73	Yes	
H3a	BAT - > PI	0.53***	8.25	0.58***	9.24	Yes	
H3b	BAT - > PP	0.33***	4.78	0.36***	5.60	Yes	
H3c	BAT - > WOM	0.41***	6.02	0.36***	5.20	Yes	
**Moderating relationships**							
H4	CPBL × CET - > BAT	0.12*	2.13	0.10	1.49	Partial	
H5	CPBG × CET - > BAT	−0.06	1.10	−0.14*	2.21	Partial	
**Control variable (brand familiarity)**							
BF - > BAT		0.46***	6.34	0.16*	2.13	Yes	
BF - > PI		0.33***	4.39	0.10	1.82	Partial	
BF - > PP		0.26**	3.19	0.20**	3.10	Yes	
BF - > WOM		0.24***	3.34	0.15*	2.11	Yes	
**Mediating relationships**		**Indirect**	**Direct (β)**	**Med.**	**Indirect**	**Direct (β)**	**Med.**
H6a CPBL - > BAT - > PI		0.19***	N. S (−0.06)	Full	0.20***	S (0.13**)	Partial
H6b CPBL - > BAT - > PP		0.12***	N. S (0.03)	Full	0.12***	S (0.14**)	Partial
H6c CPBL - > BAT - > WOM		0.15***	N. S (0.04)	Full	0.12***	S (0.25***)	Partial
H7a CPBG - > BAT - > PI		0.14***	N. S (0.04)	Full	0.19***	S (0.14**)	Partial
H7b CPBG - > BAT - > PP		0.09**	S (0.15*)	Partial	0.12***	S (0.23***)	Partial
H7c CPBG - > BAT - > WOM		0.11**	S (0.18**)	Partial	0.11***	S (0.20**)	Partial

t > 1.96 at *p < 0.05; t > 2.58 at **p < 0.01; t > 3.29 at ***p < 0.001; (two-tailed); S represent significant, and N. S as Non-significant.

Finally, the permutation test results revealed no significant differences (with a *p*-value greater than 0.05) in all hypothesized relationships between China and Pakistan. Therefore, it was demonstrated that consumers from two cultures perceived and assessed the identical brand attribute indicators in a similar way. However, the magnitudes of the effects varied according to the consumers’ preferences and lifestyles.

Chin (1998) proposed that the *R*^2^ values of 0.19, 0.33, and 0.67 correspond to weak, moderate, and strong explained variance effects on endogenous constructs. Table 7 explains the strong variance effects in Pakistan but moderate to strong effects in China. To test the model fit, the SRMR is commonly used in PLS-SEM (Hair Joseph et al., 2019). The results fulfilled the SRMR (<0.08) criterion (Hu and Bentler, 1998) in both emerging markets. The predictive relevance (*Q*^2^) values 0.50, 0.25, and 0 represent the theoretical model’s high, medium, and small predictive accuracy (Hair et al., 2018). This study used the multi-group blindfolding procedure to determine predictive accuracy (Geisser, 1974) and found that model had medium to strong predictive accuracy in China and Pakistan (see Table 7). As a result, the proposed model has good predictive relevance in both emerging markets.

**TABLE 7:** *Q*^2^, *R*^2^, and SRMR values (results).

Construct	China		Pakistan	
	*Q* ^2^	*R* ^2^	*Q* ^2^	*R* ^2^
BAT	0.40	0.68	0.43	0.68
PI	0.40	0.67	0.46	0.74
PP	0.29	0.46	0.42	0.68
WOM	0.34	0.57	0.41	0.71
SRMR value	0.03		0.02	

## Discussion

This research revealed intriguing findings such as Hypothesis H1–H2 demonstrated that CPBL and CPBG positively impacted brand attitude in both emerging markets. However, CPBL had a greater influence in China than in Pakistan, whereas CPBG had a greater influence in Pakistan than in China. The findings corroborated Halkias et al. (2016), who found that brand localness and globalness perceptions positively influenced consumers’ brand attitudes in developed markets (Austria). Thus, our findings contribute to new perspectives by demonstrating that consumer perceptions of brand localness favorably impacted brand attitude in emerging markets but with a greater influence in China compared to Pakistan. The impacts of CPBL on brand attitude may be greater in China (than in Pakistan) because Chinese consumers have a strong affinity with local brands due to their perceived prestige and expressiveness of brand identity (Xie et al., 2015). On the other side, consumer perceptions of brand globalness favorably impacted brand attitude in emerging markets but with a higher influence in Pakistan than China. The effects of CPBG on brand attitude may be more pronounced in Pakistan (compared to China) because Pakistani consumers value global brands due to their higher perceived quality and great prestige (Akram et al., 2011).

According to H3a–H3c, brand attitude positively influenced consumer behavioral intentions (PI, PP, WOM) in both emerging markets, with PI and PP having greater effects in Pakistan than in China and WOM having a greater effect in China than in Pakistan. Prior research validated our findings that consumer attitudes toward brands can influence consumer behavioral intentions, such as PI in developed markets (Austria) and emerging markets (Mexico) (Halkias et al., 2016; Lopez-Lomelí et al., 2019b) and PP in developed markets (Austria) (Davvetas et al., 2015). Thus, our findings showed that brand attitude strongly influenced PI and PP in Pakistan than in China. The literature on the positive influence of brand attitude on consumers’ WOM is lacking in the CPBL and CPBG contexts. As a result, this study provides a better understanding of the favorable impact of brand attitude on developing consumers’ positive WOM more efficiently in China than in Pakistan.

**FIGURE 2:**
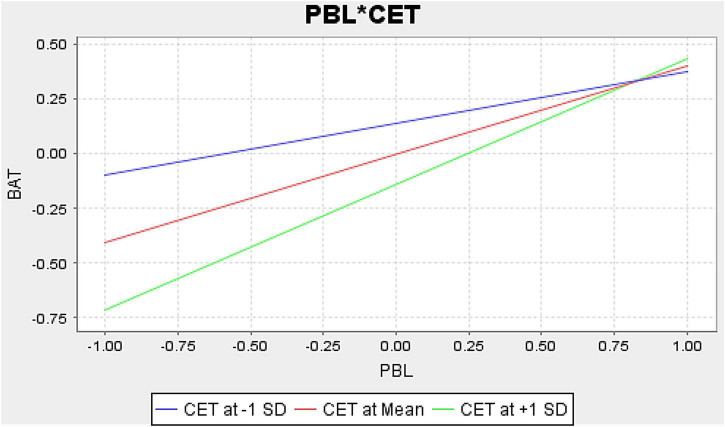
Hypothesis H4 consumer ethnocentrism moderation graph (China).

H4 revealed that the interaction effects of CET and CPBL positively influenced brand attitude in China, but no effects were observed in Pakistan. Although CET directly influenced brand attitude in Pakistan, but it dampened the influences of CPBL on brand attitude in Pakistan. Thus, it demonstrates that Chinese ethnocentric consumers prefer local brands to strengthen their economy and employment opportunities. The following Figure 2 revealed that high ethnocentric consumers strengthened the relationships of CPBL and its impact on brand attitude and lessened the impact for low ethnocentric consumers.

The findings were consistent with the earlier research by Shimp and Sharma (1987), who initially discussed the concept of consumer ethnocentrism in the American context that ethnocentric consumers prefer to buy local products and discourage from buying imported products because they think that using local products is a patriotic act. It is helpful to support the domestic economy. Similarly, Steenkamp et al. (2003) discussed that high ethnocentric American consumers were inextricably linked to local brands due to their affinity with local culture and concerns about the domestic economy. Thus, our findings contribute to ethnocentric consumers’ positive attitudes toward perceived local brands in China’s perspective.

**FIGURE 3:**
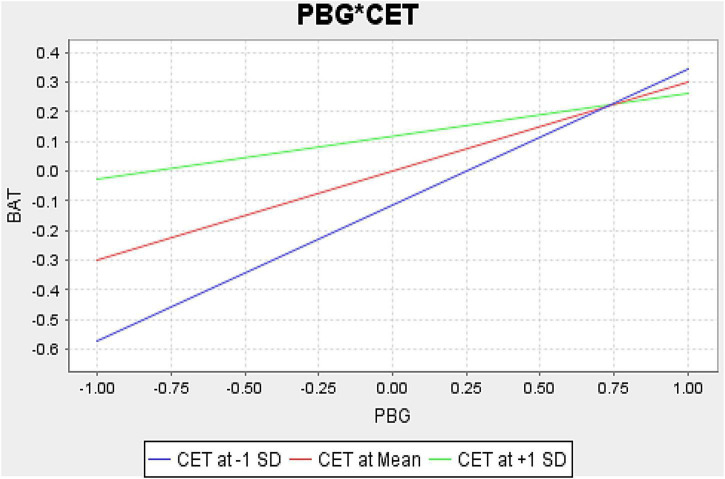
Hypothesis H5 consumer ethnocentrism moderation graph (Pakistan).

H5 revealed that the interaction moderating effects of CET and CPBG on brand attitude were negatively significant in the Pakistani environment. These findings were followed by Alden et al. (2006), who discovered that CET was negatively significant in Korean consumer attitudes toward global brands. In contrast, the same moderating effects were negatively non-significant on brand attitude in China. These findings followed the past research of Batra et al. (2000), who found that CET had a negative non-significant moderating impact on the effects of perceived non-local origin brands on consumer brand attitude in (the Indian) emerging market, and Davvetas et al. (2015) also found the similar findings in (Austria) developed market. The following Figure 3 indicates that CET’s negative mean value between the relationship of CPBG and brand attitude strengthened the significant negative effects of high ethnocentric and lessened the effects for low ethnocentric Pakistani consumers. According to Naseem et al. (2015), ethnocentric consumers feel proud to use their own country’s products because they think foreign products are a big threat to their domestic economy. Thus, these contrary findings of CET may require more research in emerging markets context.

H6a–H6c demonstrated that brand attitude was a critical mediator, particularly in China’s (vs. Pakistan) environment, as full mediation was observed between CPBL and consumer behavioral intentions (PI, PP, WOM). However, there was partial mediation between CPBL and consumer behavioral intentions (PI, PP, WOM) in Pakistan. Thus, these findings demonstrated that consumer attitudes toward perceived local brands are important in emerging markets. H7a–H7c discovered that brand attitude had a significant mediating function between CPBG and consumer behavioral intentions (PI, PP, and WOM) in both emerging markets. The findings indicated that brand attitude functioned as a partial mediator in both emerging markets, except for purchase intention in China. Thus, these findings revealed that consumer attitudes toward perceived global brands are essential in emerging markets.

## Conclusion

This research concluded that CPBL and CPBG significantly improved brand attitude, which in turn positively influenced consumer behavioral intentions (PI, PP, WOM) in China and Pakistan. Specifically, CPBL had substantial effects on brand attitude in China, while CPBG had substantial effects on brand attitude in Pakistan. Further, the brand attitude was revealed as a more critical mediator in China than in Pakistan. Moreover, significant interaction moderating impacts of CET and CPBL on brand attitude was reported in China, while CET and CPBG had negative interaction moderating effects on brand

attitude in Pakistan. Thus, understanding consumer attitudes and behavioral intentions can help local and multinational companies to increase their market share in emerging markets. Similarly, firms can segment consumers by following their ethnocentrism levels as emerging markets are a highly attractive segment in the world.

### Theoretical Implications

This research contributed several ways to the attitude theory (Fishbein and Ajzen, 1975). First, this study established that CPBL and CPBG had a beneficial effect on brand attitude in emerging markets (China-Pakistan). The attitude proposed that an individual’s attitude depends on the attributes or outcomes of performing a behavior, and assessing the outcomes is caused by brand evaluation. Therefore, a person who believes positively performing the behavior will likely generate valued outcomes that will positively impact their attitude (Ajzen and Fishbein, 2005). Consequently, brand factors like consumers’ perceptions of brand localness and globalness improve consumers’ brand attitudes in the Chinese and Pakistani contexts.

Second, this research found that brand attitude positively predicts consumer behavioral intentions (PI, PP, WOM) in both emerging markets. According to Lopez-Lomelí et al. (2019b), three outcomes are important: brand assessment (consumer beliefs/evaluation), its impact on attitude, and subsequent effects to predict consumer behavioral intentions. This research discovered that brand attributes (CPBL, CPBG) directly influenced consumer brand attitudes, which positively predicted favorable consumer behavioral intentions (PI, PP, WOM) in China and Pakistan. Finally, the positive mediating effect of brand attitude demonstrated that consumer beliefs/evaluations regarding local and global brands positively influence consumer attitudes, which in turn positively affect consumer behavioral intentions (Ajzen and Fishbein, 2005).

Similarly, this research also contributes to social identity theory. It was discovered that CET had a significant positive moderating effect on the link between CPBL and brand attitudes in the Chinese environment, whereas CET had a significant negative moderating effect on the relationship between CPBG and brand attitudes in the Pakistani context. Social identity theory describes why individuals have more positive attitudes toward their in-group and negative attitudes toward their out-group (Tajfel, 1979). International marketing experts have extended the social identity theory by researching German consumers’ perceptions of local brands representing the in-group and global brands representing the out-group (Loebnitz and Grunert, 2019). Thus, this study contributes to emerging markets by revealing that ethnocentric consumers have positive perceptions of local brands (in-group) in China, whereas ethnocentric consumers have negative perceptions of global brands (out-group) in Pakistan.

### Managerial Implications

This research revealed several managerial guidelines to local and global managers for effective brand positioning, market segmentation, and targeting strategies in China and Pakistan. First, this study demonstrated a highly significant influence of CPBL on brand attitude in China and a strong significant impact of CPBG on brand attitude in Pakistan. Therefore, considering the dominant effects of CPBL on brand attitude in Chinese consumers, the global managers should improve the brands’ localness through close associations with the local culture and communities to increase the local consumer base. Such local culture positioning strategies assist in developing brand localness in local markets, and association with local cultural norms and values encourage consumers to develop positive attitudes toward brands. On the other hand, increasing the impact of brand localness is a competitive edge for local managers. Local managers should improve this competitive edge by involving more local community welfare works. These practices may strengthen the brands’ localness and positively affect consumer attitudes toward local brands.

Similarly, the strong impact of CPBG on brand attitude has provided opportunities for global managers to increase their business share in the Pakistani markets. Global managers can also increase global brands’ functional and psychological benefits to strengthen their brands’ globalness and positively influence consumer attitudes toward perceived global brands. Multinational corporations can further associate their brands with global consumer cultures by adding different global campaigns or slogans, such as Panasonic displayed “A Better Life, A Better World” to improve their CPBG. On the other hand, local managers can enhance their brand’s globalness by using local brands’ authenticity, quality, and multi-market reach.

Second, the local and global managers should focus on brand attitude for shaping consumer behavioral intentions (PI, PP, WOM) in emerging markets. Thus, a friendly and positive attitude toward a product may lead to consumer buying behavior in emerging markets since local brands are categorized as local icons and ascribed to national identifications and community support. These brands are highly valued because of their expressive identity and trust. Local managers may use all these tools to strengthen their brands to influence consumers’ attitudes and their subsequent impacts on consumers’ behavior to stimulate their PI, PP, and WOM in local markets. Similarly, global managers may position their brands to increase the impact of brand globalness through the brand’s superior quality and prestige.

Third, the interaction moderating effects of CET and CPBL favorably influenced brand attitude in China. Thus, global managers can concentrate their efforts on target markets by using high and low ethnocentric consumer segmentation. Likewise, local managers may benefit from authenticity, nationalism, and local cultural associations to retain high ethnocentric consumers. Similarly, by improving the brand’s quality and social value and by leveraging celebrities, local managers can foster consumers’ perceptions of the brand’s globalness and attract low ethnocentric consumers in local markets. In contrast, global managers can target and segment the low ethnocentric consumers by adding the factors of global consumer culture positioning to cultivate positive perceptions and favorable attitudes toward global brands in emerging markets (China).

Finally, this research discovered that the interaction moderating effects of CET and CPBG significantly negatively influenced brand attitude in Pakistan. The findings indicate that Pakistani ethnocentric consumers were biased toward global brands due to their strong ties to local culture and the domestic economy. Thus, local managers can segment markets into low and high ethnocentric customers by creating distinct positioning strategies to promote the adoption of local brands. In contrast, global managers can benefit by targeting and segmenting low ethnocentric consumers based on their brand’s prestige and social status in emerging markets (Pakistan).

### Limitations and Future Research Scope

There are certain limitations to this research. First, this research was restricted to two emerging markets (China-Pakistan). Recent trends revealed that consumers in developed markets prefer local brands. Therefore, future researchers may extend this topic by incorporating new relevant concepts in emerging and developed markets. Second, this research collected primary data using a non-probability convenient technique and attempted to generalize the findings using a larger sample size in emerging markets. However, we believe it is insufficient to generalize the findings because emerging markets vary in political, economic, social, legal, and cultural aspects, and there is a huge variation in ethnicities. Future research should add more countries with a larger sample size based on the probability technique, which may help generalize the findings to all emerging markets. Third, the study mainly collected data from students, and sample composition, particularly in Pakistan, was unequal. Thus, future research should collect data from various working professionals with equal sample composition (male vs. female), which may reveal new insights for contribution to the literature. Finally, we discovered that the moderating effects of consumer ethnocentrism on brand attitude in emerging markets were inconsistent with CPBL and CPBG. Such contradictory findings may require additional research to extrapolate the findings to emerging markets.

## Data Availability Statement

The raw data supporting the conclusions of this article will be made available by the authors, without undue reservation.

## Author Contributions

AS identified the research gap, developed the theoretical model, and finished the article’s write-up. YZ interpreted the results and reviewed and improved the article. MA worked on methodology and collected data. FL conducted the data analysis. All authors read and approved the final manuscript.

## Conflict of Interest

The authors declare that the research was conducted in the absence of any commercial or financial relationships that could be construed as a potential conflict of interest.

## Publisher’s Note

All claims expressed in this article are solely those of the authors and do not necessarily represent those of their affiliated organizations, or those of the publisher, the editors and the reviewers. Any product that may be evaluated in this article, or claim that may be made by its manufacturer, is not guaranteed or endorsed by the publisher.
